# Health Care Utilization by Canadian Women

**DOI:** 10.1186/1472-6874-4-S1-S33

**Published:** 2004-08-25

**Authors:** Arminée Kazanjian, Denise Morettin, Robert Cho

**Affiliations:** 1 Health Care & Epidemiology, Faculty of Medicine, The University of British Columbia, 5804 Fairview Avenue, James Mather Building, Vancouver, Canada; 2 Centre for Health Services and Policy Research, 429-2194 Health Sciences Mall, Vancouver, Canada; 3 Centre for Chronic Disease Prevention and Control, Health Canada, 120 Colonnade Rd, Ottawa, Canada

## Abstract

**Health Issues:**

While women are reported to be more frequent users of health services in Canada, differences in women's and men's health care utilization have not been fully explored. To provide an overview on women's healthcare utilization, we selected two key issues that are important for public policy purposes: access to care and patterns of utilization. These issues are examined using primarily data from the 1998/99 National Population Health Survey, complemented by the 2000 Canadian Community Health Survey and the 2001 Health Service Access Survey.

**Key Findings:**

• Women are twice as likely as men to report a regular family physician, but that proportion is very low (15.8%).

• Women report significantly shorter specialist wait times (20.9 days) than men (55.4 days) for mental health, while the reverse is true for asthma and other breathing conditions (10.8 for men, 78.8 for women).

• Reported mean wait times are significantly lower for men than for women pertaining to overall diagnostic tests: for MRI, 70.3 days for women compared to 29.1 days for men.

**Data Gaps and Recommendations:**

• Measurement of possible system bias and its implication for equitable and quality healthcare for women requires larger provincial samples of the national surveys, along with a longitudinal design.

• Either a national database on preventive services, or better alignment of provincial databases pertaining to health promotion and preventive services, is needed to facilitate data linkage with national surveys to undertake longitudinal studies that support gender based analyses.

## Background

Although it is known that women are more frequent users of health services than men in Canada,[1] the reasons for the difference in women and men's health care utilization have not been fully explored. For example, are women seen as frequent users of primary care because of the health care system structure and data that capture fee-for-service transactions but not necessarily episodes of primary and/or acute care that reflect women's experiences of illness? Complex research questions on the interactions between sex, disease, health care utilization and social roles remain largely unanswered.

The literature regarding sex differences in health services utilization is primarily disease specific (e.g. cardiovascular disease, chronic pain) reflecting the biomedical approach to investigating health and illness. "A considerable body of research on sex differences in the use of health care services has focused on differences in the way men and women seek care and, to a lesser extent, on the degree to which the diagnostic and therapeutic steps taken by physicians may vary according to the sex of the patient."[2]

Statistics Canada reports findings from the 2000 Canadian Community Health Survey (CCHS), including indicators on health services utilization. These data show that while 81.3% of the population, 12 years and older, had contact with medical doctors in the previous 12 months, 87.2% of the female population reported such contact in the same period.[3] Conversely, women and girls were much less likely to have had no contact with medical providers (12.5%) than men and boys (24.5%).

A subsequent national survey specifically examined access to health care services, identified as a key issue in current health care debates.[4] The authors argue that while information on health services utilization is a valid measure of access, it does not provide the complete picture pertaining to the choices and experiences of those accessing the system. This survey addresses issues of access in two major areas: first contact services and specialized services for those aged 15 years and older. Difficulties in access to routine care were reported by approximately 11% who accessed such care and by 18% who accessed immediate care. Difficulties were reported by approximately 20% of those who used specialized services. Types of barriers, waiting times and patients' opinion regarding acceptability of waiting times were also examined. This report did not contain an analysis by sex and gender.

Evidently, the concepts of access and utilization are often used without further delineation, or diverse definitions are used in various studies. It is, therefore, difficult to estimate valid measures of either concept: How much access is desirable remains debatable, and large variation in opinion exists on appropriate levels of utilization for population groups. Acute care is the focal point of the Canadian system, and it favours those who have the power to successfully negotiate the system. Therefore, understanding the effect of sex and gender on health care utilization and access requires an analytic framework that acknowledges these complexities.

There is a much greater expectation for women than men to present themselves for medical care or consultation. Women are dependent on the health care system to ensure, control or terminate their fertility; healthy women are expected to have a Pap smear if sexually active and a mammogram if aged 50 or older; they talk to their doctor about the risk of osteoporosis at 50 and obtain a bone density test if 65 and older. The risk of perpetuating the view that women are not only over-users of the system relative to men but also "sicker" than men is high without a thorough analysis of the "gendered" body for the use of health care resources. Major data limitations hamper our ability to include such analysis here.

In order to provide an overview of health care utilization by women, two health surveillance issues were selected that are important for public policy purposes: access to care and patterns of utilization. Our approach to women's health provides a critical lens through which to examine possible system bias that may result in health service inequities. Although the implications for health services utilization of men's and women's social and cultural roles are a key factor in understanding women's health care experiences, the exploration of factors beyond the biological remains a serious challenge for women's health surveillance.

## Methods

### Literature Review

A literature review of the major computerized bibliographic databases MEDLINE, HealthStar, EMBASE, CINAHL, PyscInfo, and Contemporary Women's Issues from 1995 onwards was conducted. Selected Canadian studies that have used previous versions of population surveys or national databases are presented in the Discussion section to provide a contextual backdrop to findings from this study.

### Data Sources

Cross-sectional data were examined primarily from the 1998/99 National Population Health Survey (NPHS). More specifically, the 1998/99 NPHS data included responses to questions about the number of times the respondent had seen a health care provider in the previous year and where the most recent contact with a medical professional took place. For an analysis of preventive health services utilization, data were examined from the 2000 Canadian Community Health Survey (CCHS) to obtain optimal information about these services (see Appendix for information on NPHS and CCHS methods).

The data have been organized in such a way as to describe sex differences in health care utilization and, where possible, to further examine these differences by combining one or more variables: age and geography. Ideally, a more in-depth exploration of gender would have been conducted, but because of limitations in sample size that analysis could not always be done.

Data from the 2001 Health Service Access Survey (HSAS) were also examined. This survey allowed further investigation of sex differences in health care utilization. HSAS variables used in this analysis include having a regular family doctor or the reasons for not having one; use of specialist services; and waiting times (see Appendix for information on HSAS data).

### Measures

#### Contact with Primary Care Provider

Respondents who had seen or talked on the telephone with a variety of health care providers about their physical, emotional or mental health in the previous 12 months were included. It was assumed that they had seen a primary care provider if they answered that they had seen a) a family doctor or a general practitioner, or, if aged less than 18, a pediatrician; b) a vision care provider (such as an optometrist or ophthalmologist; unfortunately, separating the two professions was not possible); or c) or a nurse for care or advice. Those who answered that they had seen an "other medical doctor" were excluded, because this category included practitioners who provided specialized care. Initially, the number of times a primary care provider was seen was grouped into five categories: 1, 2–4, 5–9, 10–19, 20+.

#### Place of Most Recent Contact

Place of contact with the health care provider was constructed from responses to the question Where did the most recent contact take place? The responses from the national survey were grouped into four main categories.

#### Access to First Contact Services and Specialized Services

Respondents who indicated that they had a regular family physician and those who indicated that they had seen a specialist for a new illness or condition.

### Statistical Analysis

This secondary analysis is based on data from Statistics Canada cross-sectional surveys. Frequency distributions and cross-tabulations are used to describe overall health services utilization. The data were weighted to reflect the Canadian population. In accordance with Statistics Canada guidelines, estimates that were based on a sample of fewer than 30 were suppressed because of the unreliability of the estimate. Statistical tests were conducted using weighted proportions. The statistical significance of proportions is expressed as 95% confidence intervals (CI) calculated by the bootstrap method. The statistical significance of means was tested using *t *tests, and values of *p *< 0.05 were considered statistically significant.

## Results

### Overall Utilization of Health Services

**Figure 1:**
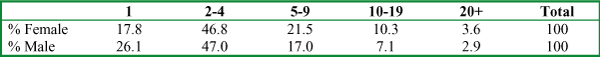
**Number of times primary care provider was seen in previous 12 months, by sex**. Statistics Canada, National Population Health Survey, 1998-99.

Overall, there is a statistically significant difference between the sexes regarding the frequency of contact with a primary care provider (as defined above) in the previous 12 months. While the most frequently reported category of health care utilization is 2 to 4 contacts for both women and men (46.8%, 47.0% respectively), women are far less likely than men to report only one health care contact (17.8%, 95% CI 16.6, 18.9 versus 26.1%, 95% CI 24.6, 27.5) and more likely to report 5 or more health care contacts in the previous year (95% CI 33.9, 36.7 versus 25.5,28.4 respectively) (Figure 1).

**Figure 2:**
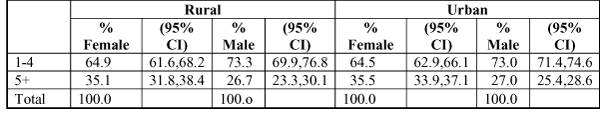
**Number of times primary care provider seen in previous 12 months, by sex and rural/urban location**. Statistics Canada, National Population Health Survey, 1998-99.

The relation between number of primary care contacts, sex and geographic location (rural/urban) was then examined (Figure 2). The table shows similarity between rural and urban frequency of contacts, reflecting higher reported frequency of contact by women regardless of location.

As expected, age is more important than sex or urban/rural location. Of the people who report having had any contact with a primary care provider in the previous 12 months, the largest proportions of high contact (5+ times) are in the age group 65 + for both rural (52.5%) and urban (53.2%) women as well as for rural (51.5%) and urban (50.7%) men.

**Figure 3:**
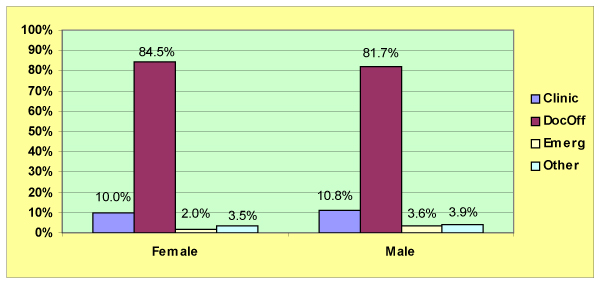
**Place of most recent primary care contact by sex**. Statistics Canada, National Population Health Survey, 1998-99.

With regard to sex differences in the location of primary care services (Figure 3), although the doctor's office is the most frequently reported place last visited by men and women (81.7% and 84.5% respectively), women are far less likely than men to have first contact in the emergency unit. The likelihood of women contacting emergency services is about half that of men (2.0%, 95% CI 1.5–2.3, versus 3.6%, 95% CI: 2.7–3.8 respectively).

### Access to Family Physicians and Specialists

**Figure 4:**
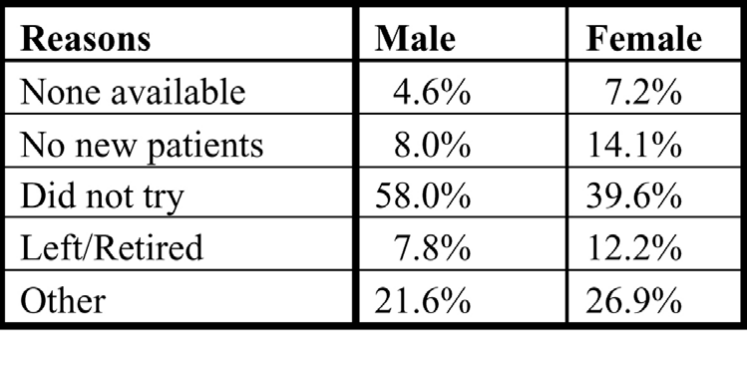
**Reasons reported for not having a regular family doctor, by sex**. Statistics Canada, Health Service Access Survey, 2001 (CCHS supplement).

Regarding issues of access, selected data from the 2001 HSAS showed that most Canadians (88%) report having a regular family doctor.[5] Men are less likely to have a regular family physician than women (15.8% of the men versus 8.8% of the women reported having no regular doctor). The reason for not having a family doctor differs between men and women (Figure 4). Men report a single main reason – they did not try to contact a family doctor – in contrast to women, who report several: they did not try to contact one, family doctors are not taking new patients or their family doctor has either left or retired.

### Utilization of Specialists by Sex, Age and Chronic Conditions

**Figure 5:**
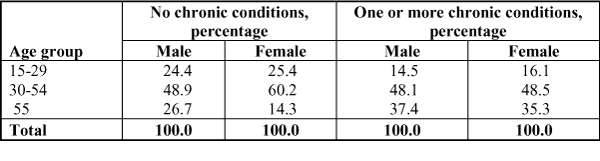
**Age distribution of those who sought specialist care, by sex and chronic condition**. Statistics Canada, Health Service Access Survey, 2001 (CCHS supplement).

Data on use of specialists, by age group, were examined, controlling for chronic conditions. Among those without chronic conditions, a higher percentage of women in the 30 to 54 year age group report seeking specialist care than men of the same age group. Distribution of specialist utilization among those with one or more chronic conditions across age is similar in men and women (Figure 5).

### Waiting Times

**Figure 6:**
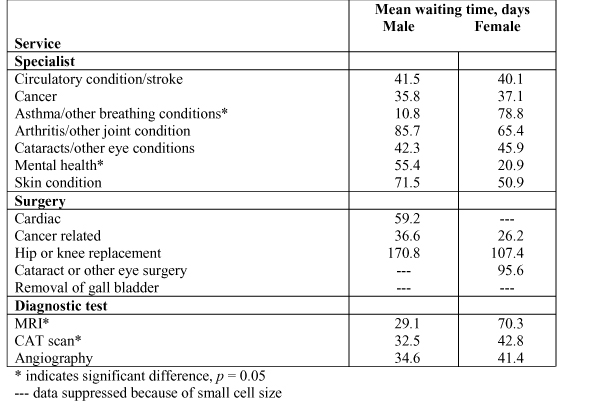
**Mean waiting time (days) for selected services, by sex**. * indicates significant difference, *p* = 0.05 --- data suppressed because of small cell size Statistics Canada, Health Service Access Survey, 2001 (CCHS supplement).

Three main categories were examined with respect to waiting times: time to specialist care, time to surgery, and time to diagnostic test. Statistically significant differences between the sexes are reported for some conditions: men wait significantly less time than women for asthma and other breathing conditions (*p *= 0.0006) but appreciably longer to see a mental health specialist (*p *= 0.035). Although some differences in wait times for surgery are reported by sex, these are not statistically significant. The mean waiting time for MRI (magnetic resonance imaging) is a great deal longer for women than for men (*p *< 0.0001), and this is also true for CAT (computerized axial tomography) scans (*p *= 0.048) (Figure 6).

### Preventive Health Services

Using the 2000 CCHS data, three types of preventive health services were examined: mammography, breast examination and Pap smear. Some differences in patterns of use by age were expected, reflecting both biological and social constructions of health and wellness. As well, individual variation in the utilization of these preventive services was anticipated, reflecting the mixed evidence on their effectiveness and the different recommendations from various clinical guidelines. Different patterns among rural residents as compared with urban residents were also expected.

#### Mammography

**Figure 7:**
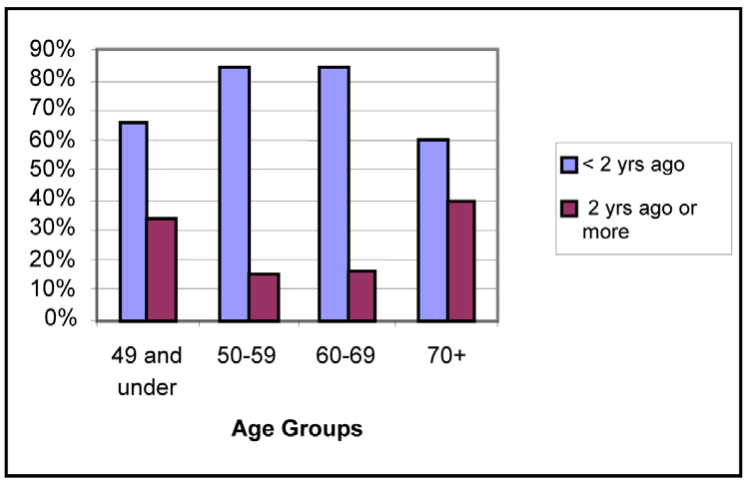
**Last time had mammography, by age group**. Statistics Canada, Canadian Community Health Survey, 2000.

The 2000 CCHS questions about mammography are addressed to women 35 years and older who have had a mammogram. For this analysis, respondents were grouped into four age groups: 49 and under, 50 to 59, 60 to 69 and 70+ (Figure 7). The two groups, 50 to 59 and 60 to 69, are very similar in recency of test, carried out less than 2 years ago. The Canadian guidelines recommend mammography for women aged 50 to 69 at 2-year intervals. This analysis shows utilization outside these guidelines among younger and older women. Age is a significant factor with regard to the last time a mammogram was received (*p *< 0.0001). However, it cannot be concluded from the survey that these are the respondents' "usual" frequency of testing.

Residency location is significant in terms of the current timing of obtaining a mammogram. Rural women are slightly more likely to report having obtained a mammogram less than 2 years ago (75.2%, 95% CI: 73.8, 76.5) than urban women (73.5%, 95% CI: 72.7, 74.4). Early information from the CCHS 2000 indicates higher overall rates of screening mammography within a year, about 70%, since the rates reported in NPHS 1996 analyses, at 63%.

#### Clinical Breast Examination (CBE)

**Figure 8:**
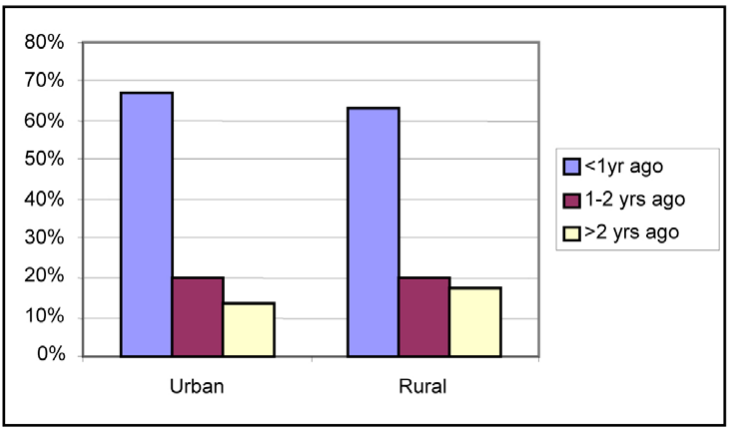
**Last time breasts were examined by doctor (rural/urban)**. Statistics Canada, Canadian Community Health Survey, 2000.

The 2000 CCHS asked female respondents 18 years of age and older questions regarding CBE by a doctor (about 65% of respondents have had a breast examination within the previous year). When asked more specifically about the last time such a procedure was done, there was a small but statistically significant (*p *< 0.0001) difference in the current timing of the examination for younger age groups (18 to 24 and 25 to 34) as compared with the older ages. In addition, there was a statistically significant difference between rural and urban residents in the reported recency of a CBE by a doctor (*p *< 0.0001). Rural women were slightly more likely (36.8%, 95% CI: 34.6, 39.0) than urban ones (32.7%, 95% CI: 31.6, 33.7) to have had a breast examination by a doctor more than 1 year ago (Figure 8).

#### Pap Smear

The 2000 CCHS data were used to determine the last time women reported having a Pap smear test, whether there was a difference between women who had had a recent Pap smear and women who had not, and whether these differences could be associated with age or geographic location. The Canadian guidelines suggest that all sexually active women be tested annually until three negative Pap smears have been reported, and then tested every 3 years until age 69.

**Figure 9:**
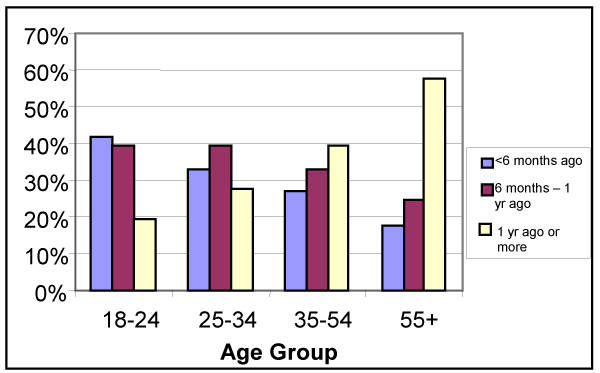
**Last time had Pap smear, by age group**. Statistics Canada, Canadian Community Health Survey, 2000.

As expected, the reported currency of Pap smear testing is inversely related to age. For example, 18 to 24 year olds are more than twice as likely (41.5%, 95% CI: 39.4, 43.6) as those 55 and older (17.6%, 95% CI: 16.7, 18.5) to report having had a test in the previous 6 months (Figure 9). Almost 60% of women aged 55+ and 40% of those 35 to 54 report having had a Pap test done more than 1 year ago. Residing in an urban area provides a small but statistically significant advantage regarding the last time a Pap smear test was done: 60% (95% CI: 59.3, 60.8) of urban residents compared with 56% (95% CI: 54.8, 57.2) of rural ones report having their test done less than a year ago.

## Discussion

### Overall Utilization of Health Services and Access

The systematic review of the literature undertaken to inform the analysis provides some interesting context to the interpretation of our findings. The results of this study are generally consistent with findings from a range of Canadian studies examining physician and hospital utilization by population groups.[2,6-9] This study has extended these observations by reporting some new findings. The approach has been to provide a general overview of women's use of health care resources across all ages to investigate possible system bias that may result in health service inequities.

Overall, the findings confirm that access to first contact with the system is generally high, in that women report a slight advantage over men. There are differences between men and women regarding reasons for not having a regular family physician and also differences regarding frequency of service utilization. While some insight is gained by examining further sex-specific utilization of specialist services for selected conditions, surgical interventions and diagnostic technologies, more complex analyses and longitudinal data are needed to delineate the relative effects of prevalence of illness, care-seeking behaviour and social roles, appropriateness of care and health outcomes by sex and gender.

### Waiting Times

Waiting times and wait lists have become the bane of the Canadian health system and related debates about a system in crisis, yet there is no general consensus about what constitutes appropriate wait times for medical and surgical care. Provincial and regional wait lists for diagnostic tests are debated in the context of fiscal policies that have not kept up with the rapid diffusion of expensive diagnostic technologies that could affect the quality of health care in Canada. Our findings indicate important differences between men and women in waiting times to receive specialist care for asthma/other breathing conditions and for mental health. It is difficult to explain the considerably longer time women have reported waiting for asthma treatment and men have reported for treatment regarding mental health problems without further analyses of respondents' health status, attitudes regarding access to health care, and other health care seeking behaviors. However, we can speculate that the reason for the large differences may be explained, at least in part, by the degree to which the diagnostic and therapeutic services provided by physicians vary according to the sex of the patient. Very little knowledge exists about this aspect of health care utilization, other than recent work on women's higher use of prescription medication for certain mental health conditions.

### Preventive Health Services and the Medicalization of Life Cycle Transitions

There is a much greater expectation for women than men to present themselves for medical care or consultation. Although women's passage through the life cycle is both a social and biological process, the focus of attention in medicine is confined to biological processes, interpreted by health care systems and providers as requiring medical management. In comparison, medical management of men occurs only in the military and sometimes when they start employment. In that context, our review of cross-sectional studies referring to earlier versions of the population surveys used in this analysis provides a historic backdrop from which to interpret our findings.

The literature alludes to increased rates of screening mammography during the last 5 to 6 years, yet the evidence is at best mixed and confounded by historic differences in the Canadian and U.S. guidelines.[10,11] An analysis of trends for 1981–94[12] traced the early implementation of breast screening program mammography across the provinces and the impact of the National Breast Screening Study on the number of mammograms during that period. That analysis used data from multiple sources: NPHS 94/95, fee-for-service data from provincial health plans and data from screening programs, where available.

While the historic data showed important provincial differences in numbers screened, this 1998/99 analysis indicates that the differences were small. More recent analyses of 1996/97 NPHS data concluded that "50% of women aged 50–69 have not had a time-appropriate mammogram".[13] When compared with the U.S. rates in 1994, the overall percentage of Canadian women reporting that they had received a mammogram in the previous year was lower: 40% and 31% respectively.[14] The screening rates were more similar in the two countries for women aged 50 to 69, partly because of consistency in clinical policies; screening was substantially higher in the United States among women aged 40 to 49 as clinical policies in Canada do not endorse screening for this age group. It would be most informative to undertake longitudinal comparative studies of U.S. and Canadian women to quantify the relative influence of the health system, clinical policy and individual care-seeking behaviour on screening mammography and health outcomes.

Using the 1987 Quebec Health Survey and linking it with fee-for service physician payment data,[15] more comprehensive analyses were undertaken to examine the contribution of health services utilization variables in a multivariate model of the recency of mammography use for women aged 50 to 59 years. The study concluded that the volume of general and gynecologic medical care, but not regularity and continuity of care, was associated with recency of mammography.

Clinical breast examination is generally reported in the literature as an important aspect of preventive health behaviour, usually associated with use of screening mammography but also with Pap smear testing. The literature suggests that physician practice behaviour can explain, in part, variations in utilization rates.[15,16] A survey of rural family physicians in Ontario, undertaken to examine sex differences in medical practice related to cervical and breast cancer screening, provides interesting findings pertaining to CBE.[17] While no physician sex differences were observed in screening mammography rates, the self-reported screening rates for Pap tests and CBE were higher among female than male physicians. The latter reported that patients asked them more frequently for a referral to another physician to perform Pap tests and CBE.

Canadian women are currently advised to have an annual Pap test "once sexually active or at age 18 with a reduction in screening frequency to every three years after two normal smears to the age of 69".[17] Maxwell et al. used the 1996/97 NPHS to determine factors important in the promotion of cervical cancer screening.[17] They found that "the estimates from the NPHS fail to indicate the dynamic nature of Pap test participation (i.e. regular, opportunistic and first time testing) and the temporal relationship between promoting factors and participation." They also noted that the NPHS is unable to provide data about women's beliefs, knowledge and attitudes regarding cancer and preventive health practices. The present analysis of the 2000 CCHS data is also limited in the conclusions that can be drawn about regular Pap testing patterns and compliance with screening recommendations.

A study from Quebec using data linkage developed logistic regression models to examine women's use of health services in relation to Pap smear use.[18] Regularity of care was the most important predictor of recency of Pap smear testing among several utilization variables. Individual characteristics, such as women's age, marital status and the presence of inflammatory diseases of the genital organs, were strong predictors that remained significant even after utilization variables were controlled for.

### Limitations of the Analysis

In the Discussion section specific observations have been made about the availability of data as well as its quality and appropriateness for comprehensive gender-relevant analysis. The analysis includes a few key aspects of health services pertinent to surveillance. Hospital utilization has not been examined because an overall analysis of volume of services would not be meaningful, and condition-specific morbidity is covered elsewhere.

Also excluded from the analyses has been the use of alternative care practitioners. This is a rapidly increasing area of utilization and traditionally associated with women's use of health resources. However, as provincial health profession statutes vary greatly in terms of their regulation and public funding, it is futile to examine such specific utilization without the appropriate sample design.

Finally, we did not examine the appropriateness of women's use of health services, from a perspective of the growing medicalization of women's life cycle transitions. That would require longitudinal data as well as richer data on the context of women's lives.

## Recommendations

• For surveillance purposes, more detailed and more comprehensive information is required that would address sex-sensitive and gender-relevant research questions; e.g. a list of providers that include a range of alternative providers/therapists, or a broader range of social, environmental, and health system indicators that affect women in different ways than they affect men.

• A longitudinal design is essential to capture causal relations between utilization, the life-course and health outcomes in order to capture more accurately, and in a richer context, the range of women's health care experiences.

• As health care is under provincial jurisdiction, larger provincial samples of the national surveys are necessary, together with a longitudinal design, in order to measure possible system bias and its implication for equitable and high-quality health care for women.

• Either a national database on preventive services or better alignment of provincial databases pertaining to health promotion and preventive services is needed to facilitate data linkage with national surveys and to undertake longitudinal studies that support gender-relevant analyses.

• Facilitation (and technical support) of data linkage between administrative databases and national surveys is essential to reduce the heavy burden of extensive longitudinal surveys, and to support the validation of survey-based measurement tools, as well as to enhance our understanding of gender and health.

## Bibliography

Advisory Committee on Women's Health SurveillanceWomen's health surveillance: a plan of action for Health Canada Ottawa (ON): Minister of Public Works and Government Services Canada1999AhmadFStewartDECamersonJIHymanIRural physicians' perspectives on cervical and breast cancer screening: a gender-based analysisJ Women's Health Gender-Based Med200152010810.1089/15246090130003958411268303Applied Research and Analysis DirectorateHealthy Canadians: A federal report on comparable health indicators2002Accessed November 11, 2002BelandFLemayABoucherMPatterns of visits to hospital-based emergency roomsSoc Sci Med19984716579972063610.1016/S0277-9536(98)00029-XBirdCERiekerPPGender matters: an integrated model for understanding men's and women's healthSoc Sci Med199948745551019063710.1016/S0277-9536(98)00402-XBlaisRMaigaAAboubacarAHow different are users and non-users of alternative medicine?Can J Public Health19978815962926035510.1007/BF03403880PMC6990173BrownADMagistrettiAIStewartDEWomen's Health in Hospital Report 2001. Preliminary studies: volume two, exploring nursing, women's health, population healthHospital report research collaborative2002Canadian Institute for Health InformationHealth care in Canada 2002 May 29, 2002Accessed June 12, 2002Canadian Institute for Health InformationHealth care in Canada 2003 May 28, 2003Accessed June 13, 2003Canadian Institute for Health InformationRate of hospitalizations continues to decline, reports Canadian Institute for Health InformationAccessed August 15, 2002Canadian Institute for Health InformationRate of hospitalizations continues to decline, reports Canadian Institute for Health Information: Table 3 hospital days and average length of stay (ALOS) for Canada, provinces and territories, 1995/96 to 2000/01 Source: Hospital Morbidity Database, CIHIAccessed August 15, 2002Canadian Institute for Health InformationRate of hospitalizations continues to decline, reports Canadian Institute for Health Information: Table 4 Hospital discharges by leading diagnoses and gender, Canada, 2000/01 Source: Hospital Morbidity Database, CIHIAccessed August 15, 2002Canadian Institute for Health InformationRate of hospitalizations continues to decline, reports Canadian Institute for Health Information: Figure 2 Age-sSpecific hospitalization rates for digestive diseases by gender, Canada, 2000/01 Source: Hospital Morbidity Database, CIHIAccessed August 15, 2002Canadian Task force on Periodic Health Examination, edsThe Canadian guide to clinical preventive health care Ottawa: Canada Communications Group199478895CooperHInvestigating socio-economic explanations for gender and ethnic inequalities in healthSoc Sci Med2002546937061199948710.1016/S0277-9536(01)00118-6De GrasseCEO'ConnorAMBouletJEdwardsNBryantHBreithauptKChanges in Canadian women's mammography rates since the implementation of mass screening programsAm J Public Health19998992791035869010.2105/ajph.89.6.927PMC1508655DunlopSCoytePCMcIsaacWSocio-economic status and the utilisation of physicians' services: results from the Canadian National Population Health SurveySoc Sci Med200051123331081747510.1016/S0277-9536(99)00424-4ErdwinsCJBuffardiLCCasperWJO' BrienASThe relationship of women's role strain to social support, role satisfaction, and self-efficacyFamily Relations: Interdisciplinary Journal of Applied Family Studies20015023023810.1111/j.1741-3729.2001.00230.xFosterJAn invitation to dialogue: clarifying the position of feminist gender theory in relation to sexual difference theoryGender and Society19991343156FriesCJMenziesKSGullible fools or desperate pragmatists? A profile of people who use rejected alternative health care providersCan J Public Health20009121791092785210.1007/BF03404275PMC6979910FuhrerRStansfeldSAChemaliJShipleyMJGender, social relations and mental health: prospective findings from an occupational cohort (Whitehall II study)Soc Sci Med19994877871004883910.1016/S0277-9536(98)00290-1GaudetteLAAltmayerCANobregaKMLeeJTrends in mammography utilization, 1981 to 1994Health Rep1996817279085118Gijsbers Van WijkCMTVan VlietKPKolkAMGender perspectives and quality of care: towards appropriate and adequate health care for womenSoc Sci Med19964370720887013510.1016/0277-9536(96)00115-3GlazierRHBadleyEMGilbertJERothmanLThe nature of increased hospital use in poor neighbourhoods: findings from a Canadian inner cityCan J Public Health200091268731098678310.1007/BF03404286PMC6979985GoelVIronKWilliamsJIEnthusiasm or uncertainty: small area variations in the use of mammography services in Ontario, CanadaJ Epidemiol Community Health19975137882932854210.1136/jech.51.4.378PMC1060504GreenCAPopeCRGender, psychosocial factors and the use of medical services: a longitudinal analysisSoc Sci Med19994810.1016/s0277-9536(98)00440-710369437HallNTaking policy action to reduce benzodiazepines use and promote self-care among seniorsJ Appl Gerontol19981731851Health CanadaMammography and women's health PDF versionAccessed August 13, 2002HoferTPKatzSJHealthy behaviors among women in the United States and Ontario: the effect on use of preventive careAm J Public Health19968617559900313310.2105/ajph.86.12.1755PMC1380729HouleLGSalmoniAWPongRWLaflammeSViverais-DreslerGAPredictors of family physician use among older residents of Ontario and an analysis of the Andersen-Newman behavior modelCan J Aging20012023349IronKGoelVSex differences in the factors related to hospital utilization: results from the 1990 Ontario Health SurveyJ Womens Health1998735969958091610.1089/jwh.1998.7.359JoungIMAVan der MeerJBWMackenbachJPMarital status and health care utilizationInt J Epidemiol19952456975767289810.1093/ije/24.3.569KatzSJHoferTPManningWGHospital utilization in Ontario and the United States: the impact of socioeconomic status and health statusCan J Public Health19968725368870304KatzSJZemencukJKHoferTPBreast Cancer Screening in the United States and Canada, 1994: socioeconomic gradients persistAm J Public Health2000907998031080043510.2105/ajph.90.5.799PMC1446215KazanjianAUnderstanding women's health through data development and data linkage: implications for research and policyCan Med Assoc J1998159342459732713PMC1229594KazanjianASavoieIMorettinDHealth care utilization and gender: a pilot study using the BC Linked health data Vancouver (BC): BC Centre of Excellence for Women's Health2001KopecJAWilliamsJIToTAustinPCMeasuring population health: correlates of the health utilities index among English and French CanadiansCan J Public Health200091465701120074110.1007/BF03404831PMC6979995Ontario MDs ignoring Pap guidelinesMedical Post38July 2, 2002MaxwellCJBancejCMSniderJPredictors of mammography use among Canadian women aged 50–69: findings from the 1996/97 National Population Health SurveyCan Med Assoc J20011643293711232132PMC80725McCuskerJCardinSBellavanceFBelzileEReturn to the emergency department among elders: patterns and predictorsAcad Emerg Med20007249591073083210.1111/j.1553-2712.2000.tb01070.xMcCuskerJHealeyEBellavanceFConnollyBPredictors of repeat emergency department visits by eldersAcad Emerg Med1997458188918919110.1111/j.1553-2712.1997.tb03582.xMcDonoughPWaltersVGender and health: reassessing patterns and explanationsSoc Sci Med200152547591120665210.1016/S0277-9536(00)00159-3MercerSLGoelVFactors associated with the use of mammography: the Ontario Health SurveyCancer Prev Control19971144519765738MittmannNTrakasKRisebroughNLiuBAUtility scores for chronic conditions in a community-dwelling populationPharmacoeconomics199915369761053795510.2165/00019053-199915040-00004MittraIBaumMThorntonHHoughtonJIs clinical breast examination an acceptable alternative to mammographic screening?BMJ2000321107131105318510.1136/bmj.321.7268.1071PMC1118853MortonAMLoosCDoes universal health care coverage mean universal accessibility? Examining the Canadian experience of poor, prenatal womenWomen's Health Issues1995513942754949210.1016/1049-3867(95)00037-5MustardCADerksenSTatarynDIntensive use of mental health careCan J Psychiatry19964193101870596910.1177/070674379604100206MustardCAKaufertPKozyrskyjAMayerTSex differences in the use of health care servicesN Engl J Med1998338167883961426010.1056/NEJM199806043382307National Cancer InstituteWorking guidelines for early detection: rationale and supporting evidence to decrease mortality1987Bethesda, MD: National Cancer Institute Press OfficePotvinLCamirandJBelandFPatterns of health services utilization and mammography use among women aged 50 to 59 years in the Quebec Medicare systemMed Care19953351530773927510.1097/00005650-199505000-00006Quarterly indexImpact of age and gender on use of acute care hospitalsHosp Q199817610345315RandhawaJRileyRTrends in hospital utilization, 1982–83 to 1992–93Health Rep19957419, 46–537578996RhodesAEGoeringPNToTWilliamsJIGender and outpatient mental health service useSoc Sci Med2002541101182067310.1016/S0277-9536(01)00002-8SanmartinCHouleCTremblaySBerthelotJChanges in unmet health care needsHealth Reports20021312743957SavoieIKazanjianAUtilization of lipid-lowering drugs in men and women: a reflection of the research evidence?J Clin Epidemiol200255951011178112710.1016/S0895-4356(01)00436-XShepsSBReidRJBarerMLKruegerHMcGrailKMGreenBHospital downsizing and trends in health care use among elderly people in British ColumbiaCan Med Assoc J200016339740110976254PMC80372Statistics CanadaHealth indicators, May 2002 Catalogue no 82-221-XIE2002Statistics CanadaAccess to health care services in Canada 2001 Catalogue no 82-575-XIE, Minister of Industry2002Statistics CanadaHealth reports: How healthy are Canadians? 2001 Annual Report Catalogue no 82-003-XPE Minister of Industry2001Statistics CanadaHealth care services – recent trends [erratum appears in Health Rep 2000;11(4):63] Health Rep19999111210779929SzafranOBellNUse of walk-in clinics by rural and urban patientsCan Fam Physician2000461141910660793PMC1987669TaggartKCanadian data collection must improveMedical Post200238TudiverFFuller-ThomsonEWho has screening mammography? Results from the 1994–1995 National Population Health SurveyCan Fam Physician1999451901710463090PMC2328187TullyPMohlCOlder residents of health care institutionsHealth Rep1995727308652801TuokkoHMacCourtPHeathYHome alone with dementiaAging & Mental Health1999321710.1080/13607869956406WaldronIWeissCCHughesMEInteracting effects of multiple roles on women's healthJ Health Soc Behavior199839216369785695WangPPBadleyEMAnnual visits to GPs by elderly patients [erratum appears in Can Med Assoc J 1998;158(7):871]Can Med Assoc J19981582993009484249PMC1228822WeirRBrowneGTunksEGafniARobertsJGender differences in psychosocial adjustment to chronic pain and expenditures for health care services usedClin J Pain199627790896987310.1097/00002508-199612000-00007WilesJRosenbergMW'Gentle caring experience'. Seeking alternative health care in CanadaHealth Place20017209241143925610.1016/S1353-8292(01)00011-9WilkinsKParkECharacteristics of hospital usersHealth Rep1997927369474505
